# Are we measuring what we think we are measuring? The construct validity of a multi‐domain cognitive test battery in older Kenyan adults

**DOI:** 10.1002/alz70857_103989

**Published:** 2025-12-24

**Authors:** Rachel W Maina, Wambui Karanja, Chinedu Udeh‐Momoh, Kevin Kipkoech, Cynthia Isabel Smith, Sarah McDonagh, Shireen Javandel, Elena Tsoy, Victor Valcour, Karen Blackmon

**Affiliations:** ^1^ Brain and Mind Institute, Aga Khan University, Nairobi, Nairobi, Kenya; ^2^ SNF Global Center for Child and Adolescent Mental Health, Child Mind Institute, New York, NY, USA; ^3^ Brain and Mind Institute, Aga Khan University, Nairobi, Kenya; ^4^ Wake Forest University, School of Medicine, Winston‐Salem, NC, USA; ^5^ Memory and Aging Center, University of California San Francisco, San Francisco, CA, USA; ^6^ Global Brain Health Institute, University of California, San Francisco, San Francisco, CA, USA; ^7^ Dartmouth Hitchcock Medical Center, Lebanon, NH, USA

## Abstract

**Background:**

Transcultural adaptation and psychometric validation of cognitive assessment tools developed in high‐income countries (HICs) is essential for fair use in low‐ and middle‐income countries (LMICs). This study aims to evaluate the factor structure of a multi‐domain, multi‐modal (paper/pencil & digital) cognitive test battery and whether it conforms to globally recognized neuropsychological domains in cognitively unimpaired (CU) multilingual Kenyan adults.

**Method:**

A neuropsychological test battery was administered to an initial sample of 135 CU adults [36% males; mean (sd) age: 55 (7) y, range: 44‐ 79 y] and an independent validation sample of 218 CU adults (42% males; mean (sd) age: 52 (11) y, range: 35‐81 y). Exploratory factor analysis (EFA) was conducted in the initial sample using Principal Axis Factoring (PA) with oblique rotation (Promax) and confirmatory factor analysis was conducted in the independent sample. Input features were aligned across samples and included subtest scores from classic word list learning, story recall, and semantic fluency tests (NIH HCAP battery), as well as novel tablet‐based tests (Tablet‐based Cognitive Assessment Tool [TabCAT] Flanker, Match, Set‐Shifting, Birdwatch).

**Result:**

EFA revealed a three‐factor structure that explained 62.6% of the total variance. Episodic memory subtests loaded strongly on Factors 1 and 2. Factor 1 loadings included word learning (0.8), delayed word recall (1.0), and word recognition (0.7). Factor 2 loadings included immediate story recall (1.0) and delayed story recall (1.0). Factor 3 loadings included measures of attention, executive function, and visual associative memory from TabCAT (Match: 0.8, Set‐shifting: 0.9, Flanker: 0.5, and Birdwatch: 0.4). The language variable demonstrated very weak factor loadings across all 3 factors (PA1 = 0.1, PA2 = 0.2, PA3 = 0.3). The CFA showed moderate model fit, after adding one covariance between delayed story recall and word recognition, as per modification indices (χ 2 = 62.72, DF = 23, N = 218, p < .001, RMSEA = .089, CFI = . 963, TLI = .943, SRMR = .055).

**Conclusion:**

Findings support the construct validity of a multi‐domain cognitive test battery in Kenyan adults, although the low loading of semantic fluency suggests the need for further modification to enrich the language domain in this multilingual population.

